# Mutating the Conserved Q-loop Glutamine 1291 Selectively Disrupts Adenylate Kinase-dependent Channel Gating of the ATP-binding Cassette (ABC) Adenylate Kinase Cystic Fibrosis Transmembrane Conductance Regulator (CFTR) and Reduces Channel Function in Primary Human Airway Epithelia*

**DOI:** 10.1074/jbc.M114.611616

**Published:** 2015-04-17

**Authors:** Qian Dong, Sarah E. Ernst, Lynda S. Ostedgaard, Viral S. Shah, Amanda R. Ver Heul, Michael J. Welsh, Christoph O. Randak

**Affiliations:** From the ‡ Stead Family Department of Pediatrics,; the § Department of Internal Medicine,; the ‖ Department of Molecular Physiology and Biophysics, and; the ** Medical Scientist Training Program, University of Iowa, Iowa City, Iowa 52242 and; the ¶ Howard Hughes Medical Institute, Iowa City, Iowa 52242

**Keywords:** ABC transporter, adenylate kinase (ADK), AMP, ATP, chloride channel, cystic fibrosis, cystic fibrosis transmembrane conductance regulator (CFTR), epithelium, patch clamp, photoaffinity labeling

## Abstract

The ATP-binding cassette (ABC) transporter cystic fibrosis transmembrane conductance regulator (CFTR) and two other non-membrane-bound ABC proteins, Rad50 and a structural maintenance of chromosome (SMC) protein, exhibit adenylate kinase activity in the presence of physiologic concentrations of ATP and AMP or ADP (ATP + AMP ⇆ 2 ADP). The crystal structure of the nucleotide-binding domain of an SMC protein in complex with the adenylate kinase bisubstrate inhibitor P^1^,P^5^-di(adenosine-5′) pentaphosphate (Ap_5_A) suggests that AMP binds to the conserved Q-loop glutamine during the adenylate kinase reaction. Therefore, we hypothesized that mutating the corresponding residue in CFTR, Gln-1291, selectively disrupts adenylate kinase-dependent channel gating at physiologic nucleotide concentrations. We found that substituting Gln-1291 with bulky side-chain amino acids abolished the effects of Ap_5_A, AMP, and adenosine 5′-monophosphoramidate on CFTR channel function. 8-Azidoadenosine 5′-monophosphate photolabeling of the AMP-binding site and adenylate kinase activity were disrupted in Q1291F CFTR. The Gln-1291 mutations did not alter the potency of ATP at stimulating current or ATP-dependent gating when ATP was the only nucleotide present. However, when physiologic concentrations of ADP and AMP were added, adenylate kinase-deficient Q1291F channels opened significantly less than wild type. Consistent with this result, we found that Q1291F CFTR displayed significantly reduced Cl^−^ channel function in well differentiated primary human airway epithelia. These results indicate that a highly conserved residue of an ABC transporter plays an important role in adenylate kinase-dependent CFTR gating. Furthermore, the results suggest that adenylate kinase activity is important for normal CFTR channel function in airway epithelia.

## Teaser

**Background:** Cystic fibrosis transmembrane conductance regulator (CFTR) channels have ATPase and adenylate kinase activity.

**Results:** Mutating Gln-1291 disrupts adenylate kinase- but not ATPase-dependent gating, and it reduces channel activity in airway epithelia.

**Conclusion:** Normal CFTR function in airway epithelia may depend on its adenylate kinase activity.

**Significance:** Adenylate kinase activity is important for the function of an ATP-binding cassette transporter.

## Introduction

Cystic fibrosis transmembrane conductance regulator (CFTR)^3^ is an anion channel in the adenosine 5′-triphosphate (ATP)-binding cassette (ABC) transporter protein family (1–3). ABC proteins are defined by two nucleotide-binding domains (NBDs) that contain a conserved sequence, the ATP-binding cassette. The “cassette” comprises several highly conserved motifs, the Walker A and B motifs (4), the LSGGQ or ABC signature sequence (5–7), and the so-called D-, H-, and Q-loops (8–11). The three-dimensional structure of an ABC-NBD shows two lobes forming an “L” shape. One of the lobes (lobe I) is of α-helical and β-sheet structure and contains the phosphate-binding loop (Walker A motif), whereas the other lobe (lobe II) is predominantly α-helical and includes the ABC signature sequence (12).

The two NBDs dimerize in a “head-to-tail” orientation by binding two molecules of ATP at the dimer interface. Each ATP molecule interacts with the phosphate-binding loop of one NBD and the ABC signature motif of the other NBD (10, 13, 14). Most ABC proteins are ATPases (ATP + H_2_O → ADP + P_i_). ATP hydrolysis is orchestrated by residues from the Walker B motif and the D- and H-loops (11).

In addition to ATPase activity, CFTR (15–17) and two other non-membrane-bound ABC proteins, the DNA repair enzyme Rad50 (18) and a structural maintenance of chromosome (SMC) protein (19), also bind adenosine 5′-monophosphate (AMP) and display adenylate kinase activity. Adenylate kinases are enzymes with separate binding sites for ATP and AMP. They catalyze both the transfer of the ATP γ-phosphate onto AMP, producing two molecules of adenosine 5′-diphosphate (ADP), and the reverse reaction (ATP + AMP ⇆ 2 ADP) (20–23).

**FIGURE 1.**
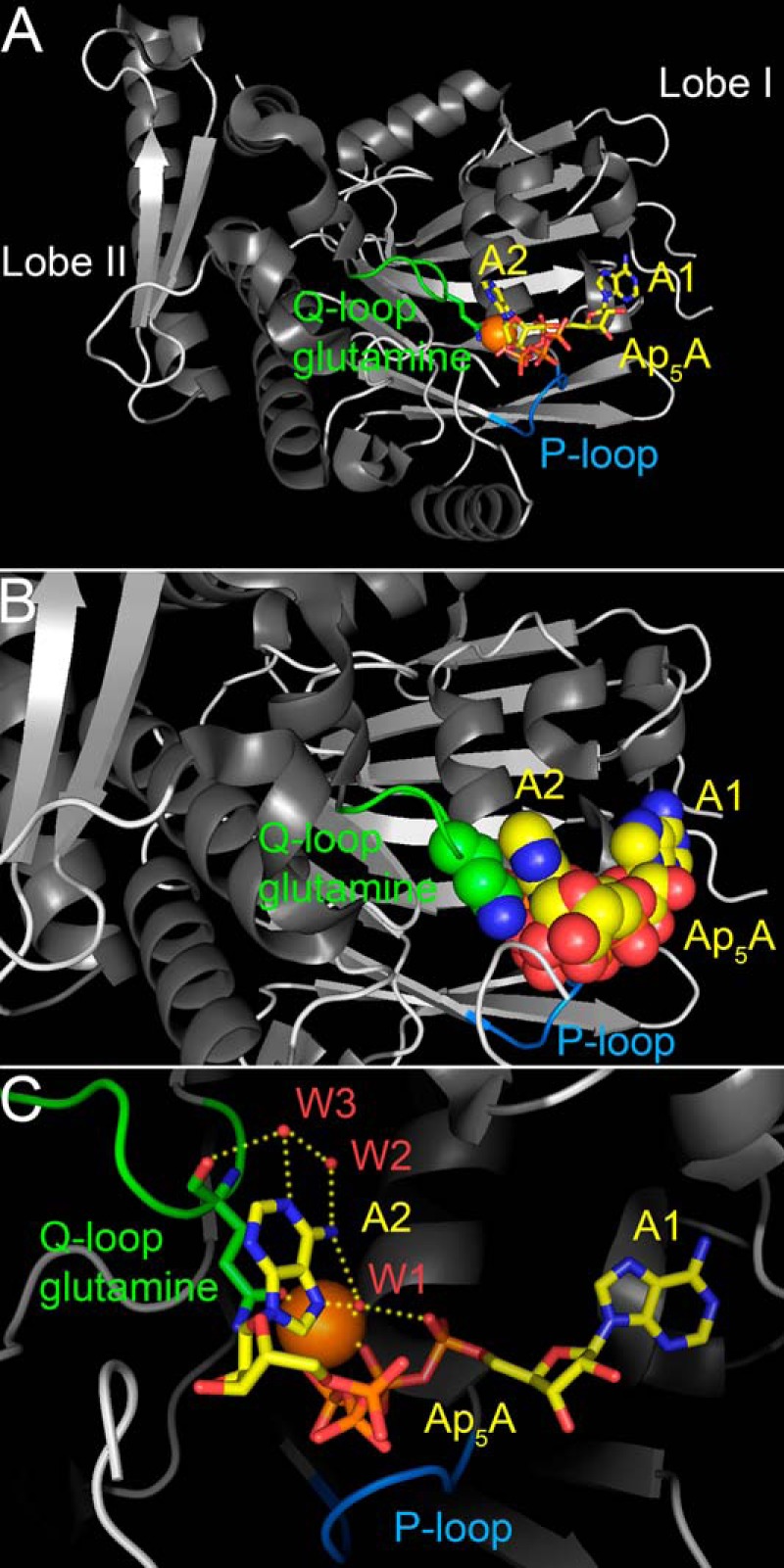
**Structure of the NBD of the *P. furiosus* SMC protein in complex with Ap_5_A (Protein Data Bank code 3KTA) (19).** *A*, one adenosine moiety of Ap_5_A (*A1*), the adjacent three phosphates, and a Mg^2+^ ion (*orange sphere*) are bound like Mg-ATP in complex with this SMC-NBD (71). The P-loop (Walker A motif) plays an essential role in phosphate group binding. The Q-loop and the position of the side chain of its conserved glutamine (Gln-145 of SMC) in relationship to the second adenosine moiety of Ap_5_A (*A2*) are depicted in *green*. The side-chain oxygen, which participates in Mg^2+^ coordination, is depicted in *red*, and the nitrogen is shown in *blue. B*, *close-up view*. The Q-loop glutamine side chain and the Ap_5_A molecule are shown in a *space-filling representation* to illustrate how the second adenosine moiety of Ap_5_A (*A2*) stacks onto the glutamine side chain. Water molecules contained in the crystal structure are not shown. *C*, several hydrogen bonds (*yellow dotted lines*) between Gln-145, the second adenosine moiety of Ap_5_A, and three water molecules (*W1*, *W2*, and *W3*; *red spheres*) are thought to confer specific recognition of adenine (19). Water-mediated base specificity has previously been observed in other nucleotide monophosphate kinases (85, 86).

Both the ATPase and adenylate kinase activities of CFTR can gate the channel. When ATP is the only nucleotide present, ATPase activity gates the channel. Evidence for ATPase-dependent gating includes effects of non-hydrolyzable ATP analogues and of mutations that disrupt this enzymatic activity (24–27). When AMP is present as well, adenylate kinase activity (ATP:AMP phosphotransfer) can also gate the channel. Evidence for adenylate kinase-dependent gating includes (*a*) effects of AMP and the adenylate kinase inhibitor P^1^,P^5^-di(adenosine-5′) pentaphosphate (Ap_5_A) and (*b*) effects of AMP analogues that cannot act as a phosphoryl group acceptor (15). CFTR interacts with AMP in an ATP-dependent manner (17). However, the CFTR amino acid residues interacting with AMP are not known. The first structural view at an AMP-binding site within an ABC protein was obtained when Lammens and Hopfner (19) solved the crystal structure of the NBD of the non-membrane-bound *Pyrococcus furiosus* SMC protein in complex with the adenylate kinase inhibitor Ap_5_A. Ap_5_A is a bisubstrate inhibitor; it contains two adenosine groups connected by five phosphate groups, allowing it to bind simultaneously to the ATP- and the AMP-binding site of an adenylate kinase (28, 29). The SMC-NBD structure shows the two Ap_5_A adenosine groups attached to two sites separated by ∼15 Å (Fig. 1). A Mg^2+^ ion, one adenosine (labeled *A1* in Fig. 1), plus α-, β-, and γ-phosphates of Ap_5_A bind the canonical Mg^2+^-ATP-binding site on lobe I of the NBD (Fig. 1*A*). The other adenosine (labeled *A2* in Fig. 1) stacks onto the side chain of the conserved Q-loop glutamine at the interface of lobe I and lobe II (Fig. 1*B*). The stacking interaction with the glutamine side-chain carboxamide group resembles an adenosine peptide bond interaction seen at the ATP-binding site. Three nitrogens of A2, the “AMP” adenosine of Ap_5_A, form hydrogen bonds to three water molecules that connect them with the main-chain carbonyl of the Q-loop glutamine and the Mg^2+^ coordination sphere. The side-chain oxygen of the Q-loop glutamine participates in coordinating the Mg^2+^ (Fig. 1*C*).

Previous work showed that Ap_5_A interacts with only one of the two ATP-binding sites of CFTR, ATP-binding site 2, and a separate AMP-binding site to inhibit enzymatic activity and current (15–17). ATP-binding site 2 comprises Walker A and B motifs of NBD2 and the ABC signature motif of NBD1 (24). The conserved Q-loop glutamine in NBD2 is Gln-1291 (9, 12, 13, 30). Based on our previous data and the SMC structure, we hypothesized that mutating Gln-1291 selectively disrupts adenylate kinase-dependent gating and causes defective channel function in the presence of physiologic concentrations of ATP, ADP, and AMP.

## Experimental Procedures

### 

#### 

##### Materials

8-N_3_-[^33^P]AMP was from Hartmann Analytic GmbH (Braunschweig, Germany). 2-N_3_-AMP was from Affinity Photoprobes, LLC (Lexington, KY). The azido-nucleotides were dissolved as triethylammonium salt in absolute methanol. Immediately before use, the methanol was evaporated under a stream of argon, and azido-nucleotides were dissolved in a buffer of 20 mm Hepes (pH 7.5), 50 mm NaCl, 3 mm MgCl_2_. [γ-^32^P]GTP, dissolved in 10 mm Tricine, pH 7.6, was from PerkinElmer Inc. Non-radioactive ATP, ADP, AMP, and Ap_5_A were from Sigma-Aldrich. ATP was used as magnesium salt. ADP, AMP, and Ap_5_A were sodium salts. Protein kinase A (PKA) catalytic subunit, purified from bovine heart, was from EMD Millipore Corp. (Billerica, MA). The monoclonal CFTR antibodies used were from R&D Systems, Inc. (Minneapolis, MN) (13-1 (31)) and EMD Millipore (Billerica, MA) (M3A7 (32) and 13-4 (33)). The 769 antibody (34) was from the University of North Carolina (Chapel Hill, NC) in conjunction with the Cystic Fibrosis Foundation. Anti-ZO-1 was from Zymed Laboratories Inc. (San Francisco, CA).

##### Site-directed Mutagenesis

Mutations into the codon for Gln-1291 were introduced into CFTR cDNA within the pTM-CFTR4 plasmid (35) using a QuikChange® II XL site-directed mutagenesis kit (Agilent Technologies, Santa Clara, CA). For each mutant, we sequenced the entire CFTR cDNA to verify its correct identity.

##### Patch Clamp Experiments

CFTR Cl^−^ currents were studied using excised, inside-out membrane patches from HeLa cells transiently expressing either wild-type or mutant CFTR using a vaccinia virus/T7 RNA polymerase expression system (36) as described previously (37, 38). When single channel studies were intended, we used instead a pcDNA^TM^3.1 (Invitrogen) CFTR expression system as described (39). The pipette (extracellular) solution contained 140 mm *N*-methyl-d-glucamine, 3 mm MgCl_2_, 5 mm CaCl_2_, 100 mm l-aspartic acid, and 10 mm Tricine, pH 7.3, with HCl. The bath (intracellular) solution contained 140 mm *N*-methyl-d-glucamine, 3 mm MgCl_2_, 1 mm CsEGTA, and 10 mm Tricine, pH 7.3, with HCl. Following patch excision, CFTR channels were activated with 25 nm PKA catalytic subunit and ATP. PKA catalytic subunit was present in all cytosolic solutions that contained ATP. Experiments were performed at room temperature (23–26 °C). Macropatch recordings were low pass-filtered at 100 Hz for analysis using an 8-pole Bessel filter (model 900, Frequency Devices, Inc., Haverhill, MA). Recordings for single channel gating analysis were low pass-filtered at 500 Hz. Data were digitized at 5 kHz. Data were analyzed as described previously (40, 41) with a burst delimiter of 20 ms using the pCLAMP software package (versions 9.2 and 10.3, Molecular Devices, Sunnyvale, CA). Events of <4 ms duration were ignored. pCLAMP software was also used for model comparisons. To determine whether increasing the number of exponential components produced a statistically better fit of the burst histogram data, an F-test was performed.

##### Ussing Chamber Studies

Well differentiated primary human bronchial airway epithelia derived from cystic fibrosis (CF) donors homozygous for the F508del mutation and cultured at the air-liquid interface (42) were infected with recombinant adenovirus serotype 5 at a multiplicity of infection of 50–100 for 1 h. The vectors encoded wild-type or Q1291F CFTR cDNA driven by a cytomegalovirus promotor. Three days after infection, epithelia were incubated overnight with 10 μm forskolin and 100 μm 3-isobutyl-2-methylxanthine (IBMX) until they were mounted in Ussing chambers. Withdrawal of chronic stimulation with cAMP agonists reduces basal CFTR activity (43). Ussing chamber experiments were performed at 37 °C as described (44) using symmetrical solutions on both surfaces of 135 mm NaCl, 2.4 mm K_2_HPO_4_, 0.6 mm KH_2_PO_4_, 1.2 mm CaCl_2_, 1.2 mm MgCl_2_, 5 mm dextrose, and 5 mm HEPES (pH 7.4) that were gassed with compressed air. We measured short circuit current and transepithelial conductance under basal conditions and after sequentially adding amiloride (100 μm), 4,4′-diisothiocyanotostilbene-2,2′-disulfonic acid (DIDS) (100 μm), forskolin (10 μm) and IBMX (100 μm), and (naphthalen-2-ylamino)-acetic acid (3,5-dibromo-2,4,-dihydroxy-benzylidene)-hydrazide (GlyH-101) (100 μm) into the apical chamber. GlyH-101 was a generous gift from Robert Bridges (Rosalind Franklin University of Medicine and Science, Chicago, IL) and Cystic Fibrosis Foundation Therapeutics.

##### Quantification of CFTR Expression

After performing Ussing chamber studies, RNA was extracted from airway epithelia following the RNeasy lipid tissue minikit (Qiagen, Valencia, CA) protocol. cDNA was prepared using a high capacity cDNA reverse transcription kit (Applied Biosystems, Foster City, CA). Primers were designed to specifically amplify GAPDH (used as reference gene) and CFTR cDNA. Primers for GAPDH were TGCACCACCAACTGCTTAGC and GGCAGGACTGTGGTCATGAG. Primers for CFTR were CTTACAAATGAATGGCATCGAAGAGG and GGTGAATGTTCTGACCTTGGTTAAC. For quantification, the Power SYBR® Green PCR master mix (Applied Biosystems, Foster City, CA) was used. Quantitative real-time PCR was performed using an Applied Biosystems® 7500 fast real-time PCR system. Δ*C_T_* values were calculated as differences between CFTR and GAPDH cycle threshold (*C_T_*) values. To quantify wild-type and Q1291F CFTR expression relative to endogenous F508del CFTR expression, the 2^−ΔΔ^*^CT^* method (45) was applied. ΔΔ*C_T_* values were the differences between the Δ*C_T_* values obtained in epithelia infected with recombinant adenovirus and uninfected epithelia from the same donor.

##### Immunocytochemistry

In some cases after performing Ussing chamber experiments (*i.e.* 4 days after gene transfer), epithelia were studied by immunochemistry as described (46). Epithelia were fixed, permeabilized, and incubated with anti-CFTR antibody 769 and anti-ZO-1 primary antibodies, followed by Alexa Fluor-conjugated secondary antibodies (Molecular Probes, Inc., Eugene, OR). Epithelia were then examined by confocal laser scanning microscopy.

##### Expression of CFTR in HeLa Cells and Preparation of Membranes for Biochemical Studies

Wild-type and mutant CFTR were transiently expressed in HeLa cells using a double vaccinia virus/T7 RNA polymerase system (36). Cell membranes were prepared as described previously (16, 17) and resuspended in 20 mm Hepes (pH 7.5), 50 mm NaCl, 3 mm MgCl_2_, 2 μg/ml leupeptin, 100 μg/ml Pefabloc, and 7 μg/ml E-64.

##### CFTR Adenylate Kinase Assay

Assays were performed as described previously (16). In brief, membranes containing 50 μg of protein were incubated gently shaking with 50 μm non-radioactive 2-N_3_-AMP, radioactive [γ-^32^P]GTP (30 μCi, 6000 Ci/mmol), 20 mm Hepes (pH 7.5), 50 mm NaCl, 3 mm MgCl_2_, and 1 mm Tricine (pH 7.6) for 5 min at 37 °C in a total volume of 30 μl followed by UV irradiation for 30 s (302 nm, 8-watt lamp) at a distance of 5 cm, as indicated in Fig. 11. Subsequently, 20 μl of stop buffer (25 mm dithiothreitol, 4% SDS, 20 mm Hepes (pH 7.5), 50 mm NaCl, 125 μg/ml benzamidine, 4 μg/ml aprotinin, 2 μg/ml leupeptin, 100 μg/ml Pefabloc, 7 μg/ml E-64) and then 875 μl of solubilization buffer (1% Triton X-100, 20 mm Hepes (pH 7.5), 50 mm NaCl, 125 μg/ml benzamidine, 4 μg/ml aprotinin, 2 μg/ml leupeptin, 100 μg/ml Pefabloc, 7 μg/ml E-64) were added. Samples were stored at −80 °C overnight. CFTR was immunoprecipitated as described (16) using monoclonal CFTR antibodies 13-1 (0.2 μg/sample) and M3A7 (1 μg/sample).

##### CFTR 8-N_3_-[^33^P]AMP Photolabeling

Photolabeling was performed, following our previously described protocol (17). Specifically, membranes containing 50 μg of protein were mixed on ice in 20 mm Hepes (pH 7.5), 50 mm NaCl, 3 mm MgCl_2_ with 8-N_3_-[^33^P]AMP (94 μCi, >3000 Ci/mmol) and 8.3 mm non-radioactive ATP, as indicated in Fig. 10, in a total volume of 30 μl. Individual reactions were immediately irradiated with UV light (302 nm, 8-watt lamp) for 30 s at a distance of 5 cm. After exposure to UV light, 20 μl of stop buffer and 875 μl of solubilization buffer were added, samples were stored at −80 °C overnight, and CFTR was immunoprecipitated as described (17).

##### Gel Electrophoresis and Autoradiography

Immunoprecipitated CFTR was fractionated on 6% SDS-polyacrylamide gels. After electrophoresis, gels were dried and then subjected to digital autoradiography using an FLA-7000 imaging system (Fuji Photo Film Co., Ltd., Tokyo, Japan). For quantitative image analysis, Multi Gauge analysis software (version 3.0; Fuji Photo Film) was used. Region intensities corresponding to CFTR bands were quantified in linear arbitrary units. Background intensities were subtracted.

##### Cell Surface Biotinylation

Wild-type and mutant CFTR were expressed in Chinese hamster ovary (CHO) cells for 48 h at 37 °C using a pcDNA^TM^3.1 (Invitrogen) expression system together with a non-liposomal low cytotoxic DNA transfection reagent (X-tremeGENE 9, Roche Applied Science). Cells were then put on ice and washed twice in phosphate-buffered saline with additional calcium and magnesium (PBS^+/+^) (138 mm NaCl, 8.1 mm Na_2_HPO_4_, 2.7 mm KCl, 1.5 mm KH_2_PO_4_, 0.9 mm CaCl_2_, 0.5 mm MgCl_2_). After a third wash in borate buffer (154 mm NaCl, 10 mm boric acid, 7.2 mm KCl, 1.8 mm CaCl_2_, pH 9.0), cells were incubated for 30 min at 4 °C in Sulfo-NHS-SS-Biotin (Thermo Fisher Scientific, Waltham, MA) dissolved in borate buffer (0.5 mg/ml). Biotinylation was quenched by washing cells three times with 100 mm glycine in PBS^+/+^, followed by two washes with PBS^+/+^ to remove glycine. Cells were then lysed in lysis buffer (50 mm Tris-HCl, pH 7.4, 150 mm NaCl, 0.1 mm PMSF, 70 μg/ml benzamidine-HCl, 10 μg/ml pepstatin A, 20 μg/ml aprotinin, 20 μg/ml leupeptin) plus 1% Nonidet P-40 (4-nonylphenyl poly(ethylene glycol)) for 40 min at 4 °C. 2.5% of the soluble cell lysate was removed for Western blotting to quantify the total amount of CFTR. The remaining 97.5% of the cell lysate were incubated with NeutrAvidin UltraLink Resin (Thermo Fisher Scientific) for 2 h at 4 °C to bind biotinylated proteins. Resins were washed extensively before bound proteins were fractionated on 6% SDS-polyacrylamide gels and analyzed by Western blotting.

##### Western Blotting

After gel electrophoresis, proteins were transferred onto a PVDF membrane (Immobilon®-FL transfer membrane, EMD Millipore, Billerica, MA). PVDF membranes blocked for 1 h in 0.1% casein were incubated for 2 h with monoclonal anti-human CFTR antibody, diluted 1:500 to 1:1000 in TTBS buffer (137 mm NaCl, 2.7 mm KCl, 25 mm Tris-Cl (pH 8.0), 0.05% Tween 20), as indicated in the figures. Membranes were washed three times in TTBS buffer and then incubated for 45 min with donkey anti-mouse IRDye® (0.05 μg/ml, in phosphate-buffered saline plus 0.1% casein, 0.01% SDS) (LI-COR Biosciences, Lincoln, NE) as secondary antibody. Membranes were washed again three times in TTBS buffer. Immunoreactive proteins were then visualized with the Odyssey Infrared Imaging System (LI-COR Biosciences) and quantified in linear arbitrary units using Multi Gauge analysis software (version 3.0; Fuji Photo Film). Background intensities were subtracted.

##### Data Presentation and Statistics

Data are presented as means ± S.E. *p* values <0.05 were considered statistically significant. For statistical analysis, SigmaStat (version 3.0; SPSS Inc., Chicago, IL) and Prism (version 6.04; GraphPad Software, Inc., La Jolla, CA) software were used.

## Results

### 

#### 

##### Gln-1291 Mutations Interfere with Ap_5_A Inhibition of CFTR Current

**FIGURE 2.**
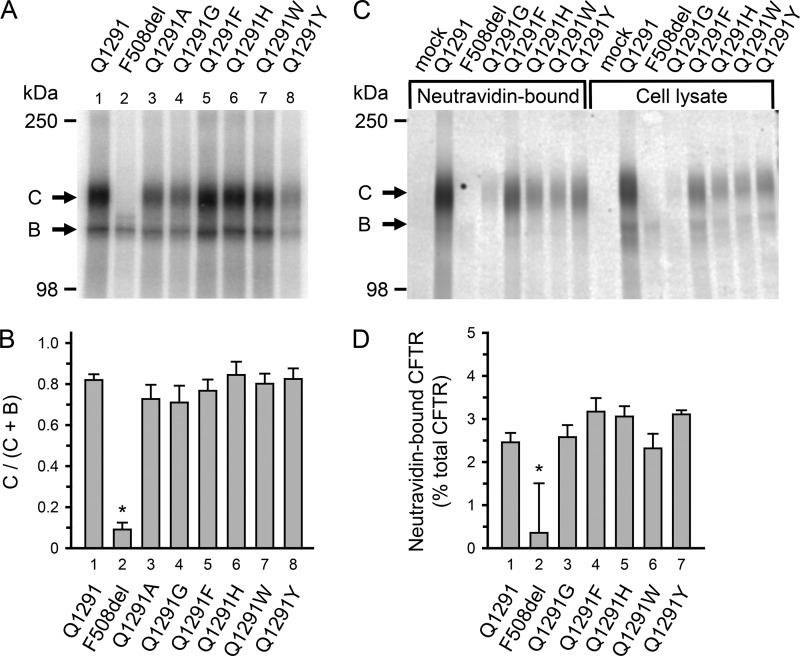
**Processing of CFTR with Gln-1291 mutations.** *A* and *B*, processing was evaluated by assessing CFTR glycosylation. CFTR core glycosylation occurs in the endoplasmic reticulum. The core-glycosylated CFTR migrates as *band B* during SDS-gel electrophoresis. Later, CFTR becomes highly glycosylated in the Golgi apparatus and then migrates as *band C*. Deletion of phenylalanine 508 (*F508del*) illustrates a mutation causing a processing defect at 37 °C; most of F508del CFTR is degraded before reaching the Golgi complex and therefore does not appear as band C (31, 35, 87, 88). *A*, autoradiograph. Wild-type and mutant CFTR were expressed in 293T cells for 48 h at 37 °C using a vaccinia virus-T7 hybrid expression system (36). CFTR was solubilized, immunoprecipitated, and phosphorylated with the catalytic subunit of protein kinase A and [γ-^32^P]ATP (38). Immunoprecipitates were fractionated on 6% SDS-polyacrylamide gels. *B*, quantitative data for the fraction of CFTR migrating as band C. Radioactivity incorporated into bands B and C was quantified by digital autoradiography. Depicted is the ratio of radioactivity in band C *versus* the total radioactivity in bands B and C. Data for wild-type (*Q1291*) CFTR and F508del CFTR are depicted for comparison and have been previously shown in the supplemental material of Ref. 17. *, *p* < 0.001 when compared with *bar 1*, *3*, *4*, *5*, *6*, *7*, or *8*. No significant differences were detected between *bars 1*, *3*, *4*, *5*, *6*, *7*, and *8* (one-way ANOVA followed by Holm-Sidak's method of all pairwise multiple comparisons; wild-type CFTR, *n* = 13; F508del CFTR, *n* = 10; Q1291A CFTR, *n* = 4; Q1291G CFTR, *n* = 4; Q1291F CFTR, *n* = 6; Q1291H CFTR, *n* = 6; Q1291W CFTR, *n* = 6; Q1291Y CFTR, *n* = 6). *C* and *D*, processing to the plasma membrane was evaluated by assessing CFTR cell surface biotinylation. Experiments were performed as described under “Experimental Procedures.” *C*, Western blot probed with CFTR antibody 769 of NeutrAvidin-bound protein from 97.5% of the total soluble cell lysate (*left eight lanes*) and 2.5% of the total cell lysate (before incubating with NeutrAvidin; *right eight lanes*). *Letters* label highly glycosylated (*C*) and core-glycosylated (*B*) CFTR. The NeutrAvidin-bound CFTR migrated almost exclusively as *band C*. Mock-transfected cells received pcDNA^TM^3.1 vector without CFTR cDNA insert. *D*, quantitative data for the fraction of CFTR (in percent) that bound to NeutrAvidin after exposing intact cells to cell surface biotinylation. CFTR protein was quantified by Western blotting as described under “Experimental Procedures.” *, *p* < 0.01 when compared with *bar 1*, *3*, *4*, *5*, *6*, or *7* (one-way ANOVA followed by Holm-Sidak's method of multiple comparisons *versus* control group; wild-type CFTR, *n* = 6; F508del CFTR, *n* = 4; Q1291G CFTR, *n* = 4; Q1291F CFTR, *n* = 6; Q1291H CFTR, *n* = 4; Q1291W CFTR, *n* = 4; Q1291Y CFTR, *n* = 4). No significant differences were detected between *bars 1*, *3*, *4*, *5*, *6*, and *7* (one-way ANOVA). *Error bars*, S.E.

**FIGURE 3.**
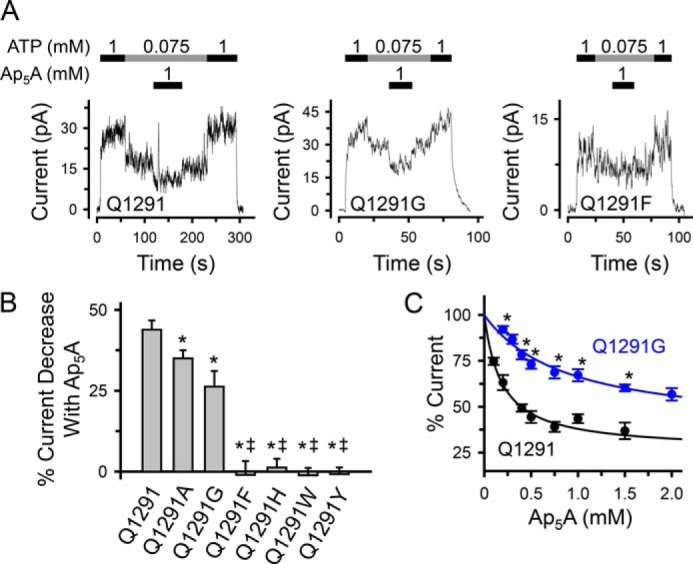
**Effect of Gln-1291 mutations on Ap_5_A inhibition of CFTR Cl^−^ current.** *A*, current recordings (100 ms averages) from excised inside-out membrane patches containing multiple wild-type (*Q1291*) or mutant CFTR channels. ATP and Ap_5_A were present during the times and at the concentrations indicated by *bars*. ATP was added together with PKA catalytic subunit as described under “Experimental Procedures.” Holding voltage was −40 mV. *B*, quantitative data. The percentage of current decrease with Ap_5_A was calculated as the difference between the current at 75 μm ATP before and after adding 1 mm Ap_5_A, divided by the current in the absence of Ap_5_A (at 75 μm ATP) and multiplied by 100. Experiments were performed as shown in *A*. Each *column* shows the mean ± S.E. (*error bars*) of 5–15 individual experiments obtained from at least two membrane patches per CFTR variant. *, *p* < 0.05 compared with wild type; *double daggers*, *p* < 0.001 compared with Q1291G (one-way ANOVA followed by Holm-Sidak's method of multiple comparisons *versus* control group). *C*, removal of the Gln-1291 side chain (Q1291G mutation) increases the apparent dissociation constant of the Ap_5_A-CFTR complex (*K_i_*_,app_). Experiments were performed as illustrated in *A* with Ap_5_A added at various concentrations in the presence of 75 μm ATP and PKA catalytic subunit. Current was normalized to the current at 75 μm ATP in the absence of Ap_5_A (100%). Data are from eight patches with wild-type CFTR and 11 patches with Q1291G CFTR; *n* ≥ 4 for each point. *Asterisks*, *p* < 0.01 compared with wild type at the same Ap_5_A concentration (Mann-Whitney rank sum test). *Lines* are fit to the equation for one Ap_5_A binding site (17), percentage inhibition (%inh) = 100 − ((%inh_max_ × [Ap_5_A])/([Ap_5_A] + *K_i_*_,app_)), and maximal inhibition (%inh_max_) of 73.7 ± 4.1% and *K_i_*_,app_ of 0.187 ± 0.033 mm for wild type (*black line*), and %inh_max_ of 63.2 ± 6.5% and *K_i_*_,app_ of 0.875 ± 0.192 mm for Q1291G CFTR (*blue line*).

Previous studies showed that Ap_5_A partially inhibited wild-type CFTR, and inhibition could be attenuated by high ATP concentrations (15). To test the hypothesis that Gln-1291 interacts with Ap_5_A, we examined the effect of different Gln-1291 mutations. We predicted that if one Ap_5_A adenosine stacked onto the side chain of the conserved Q-loop glutamine in a similar way as in the SMC crystal structure (Fig. 1*B*) (19), substitutions with bulky side chains would sterically prevent Ap_5_A binding and abolish Ap_5_A inhibition. Studies with SMC and non-ABC adenylate kinases showed that several amino acids interact with Ap_5_A (19, 29). We therefore further predicted that shortening or removal of the side chain at position 1291 might decrease Ap_5_A binding but would not abolish its interaction with CFTR. None of the Gln-1291 mutations that we used interfered with processing and trafficking of the mutant CFTR to the plasma membrane (Fig. 2). We studied wild-type and mutant CFTR in excised inside-out membrane patches using the patch clamp technique. The addition of protein kinase A, catalytic subunit, and ATP to the cytosolic surface of the patches generated channel activity. Because high ATP concentrations reduce Ap_5_A inhibition, we lowered the ATP concentration to 75 μm (Fig. 3*A*). We found that Ap_5_A did not inhibit Cl^−^ current when Gln-1291 was replaced by amino acids with bulky side chains like phenylalanine (Q1291F), tryptophan (Q1291W), tyrosine (Q1291Y), or histidine (Q1291H). Gln mutations that resulted in shortening (Q1291A) or deletion (Q1291G) of the side chain reduced Ap_5_A inhibition (Fig. 3, *A* and *B*). To further evaluate the effect of these mutations, we tested inhibition of Q1291G CFTR current with a range of Ap_5_A concentrations. We found that the apparent dissociation constant of the Ap_5_A-CFTR complex increased ∼5-fold when the side chain of Gln-1291 was removed (Fig. 3*C*). These results are consistent with a role of Gln-1291 in the interaction of CFTR with Ap_5_A.

##### Gln-1291 Substitutions with Bulky Side Chains Do Not Significantly Alter the Interaction of CFTR with ATP and ATPase-dependent Gating

**FIGURE 4.**
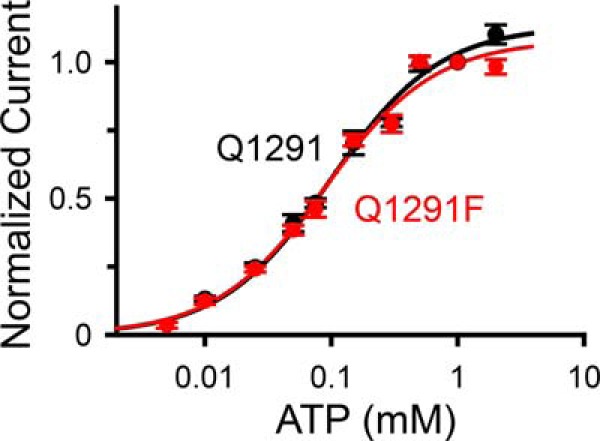
**Effect of ATP concentration on current of wild-type (*Q1291*; *black*) and Q1291F CFTR (*red*).** Experiments were performed as shown in Fig. 3*A*. Data are from two wild-type and 15 Q1291F CFTR patches with *n* ≥ 8 for each ATP concentration. Because each patch contained a different number of CFTR channels, all current measurements were normalized to the current obtained with 1 mm ATP in the same patch. *Lines* are fits to a Michaelis-Menten equation using apparent *K_m_* values of 98 ± 7 μm (wild type; *black line*) and 89 ± 6 μm (Q1291F mutant; *red line*) and maximum normalized current values at high ATP concentrations of 1.13 ± 0.02 (wild type; *black line*) and 1.08 ± 0.02 (Q1291F mutant; *red line*). *Error bars*, S.E.

Ap_5_A interacts with CFTR by binding simultaneously to ATP-binding site 2 and an AMP-binding site (15, 17). Thus, bulky side-chain substitutions at position 1291 could block the interaction of Ap_5_A either at ATP-binding site 2 or at the AMP-binding site. To test the effect of such a mutation on the interaction of CFTR with ATP and ATPase-dependent gating, we compared the relationship between current and ATP concentration of wild-type CFTR with that of Q1291F CFTR and found that they were similar (Fig. 4). This result indicates that the Q1291F mutation does not substantially alter the potency of ATP at stimulating current.

**FIGURE 5.**
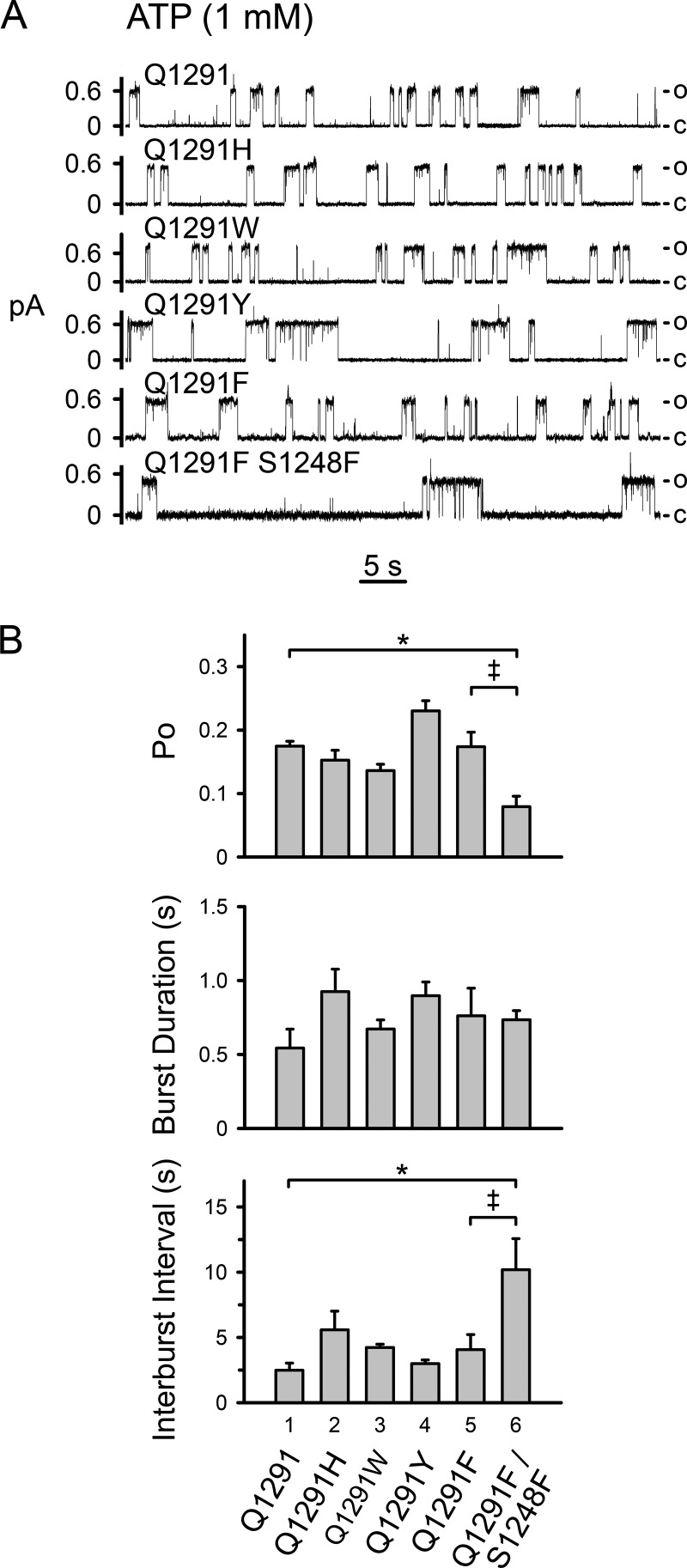
**Influence of Gln-1291 mutations on single-channel gating.** *A*, examples of 1-min current recordings from excised membrane patches containing wild-type (Q1291) or mutant CFTR in the presence of 1 mm ATP at the cytosolic surface. Holding voltage was −80 mV for wild-type, Q1291W, and Q1291Y CFTR and −60 mV for Q1291H, Q1291F, and Q1291F/S1248F CFTR. For illustration purposes, traces were digitally low pass-filtered at 50 Hz. *c*, channel closed state; *o*, single-channel open state. *B*, average single channel properties. Data are means ± S.E. (*error bars*) of 3–6 patches. *Top*, *P_o_*, open state probability. *, *p* < 0.01. No significant differences were detected between *bar 1* and *bars 2*, *3*, *4*, and *5* (one-way ANOVA followed by Holm-Sidak's method of multiple comparisons *versus* control group). *Double dagger*, *p* < 0.05 (Student's *t* test). *Middle*, mean burst duration. No significant differences were detected (one-way ANOVA). *Bottom*, mean interburst interval. *, *p* < 0.05 (Kruskal-Wallis one-way ANOVA on ranks followed by Dunn's method of multiple comparisons *versus* control group). No significant differences were detected between *bars 1*, *2*, *3*, *4*, and *5* (Kruskal-Wallis one-way ANOVA on ranks). *Double dagger*, *p* < 0.05 (Mann-Whitney rank sum test).

We also determined the influence of bulky Gln-1291 side-chain substitutions on single channel gating in the presence of ATP. These mutations did not significantly alter open probability (P*_o_*), mean burst duration, and length of the interburst intervals (Fig. 5). In contrast, previous studies examining the effect of blocking the interaction of ATP with either binding site showed that such blockage resulted in a very low channel open probability (41, 47, 48). Thus, our data suggest that the Gln-1291 mutations did not disrupt the interaction of CFTR with ATP. As a positive control, we mutated serine 1248 to phenylalanine (S1248F) in Q1291F CFTR. The S1248F mutation prevents the interaction of ATP with ATP-binding site 2 (48). As anticipated, the open probability of the double mutant was markedly reduced, mainly due to interburst closed times that were significantly longer than those of wild-type and Q1291F CFTR (compare *bar 6* with *bars 1* and *5* in the *top* and *bottom panels* of Fig. 5*B*).

**FIGURE 6.**
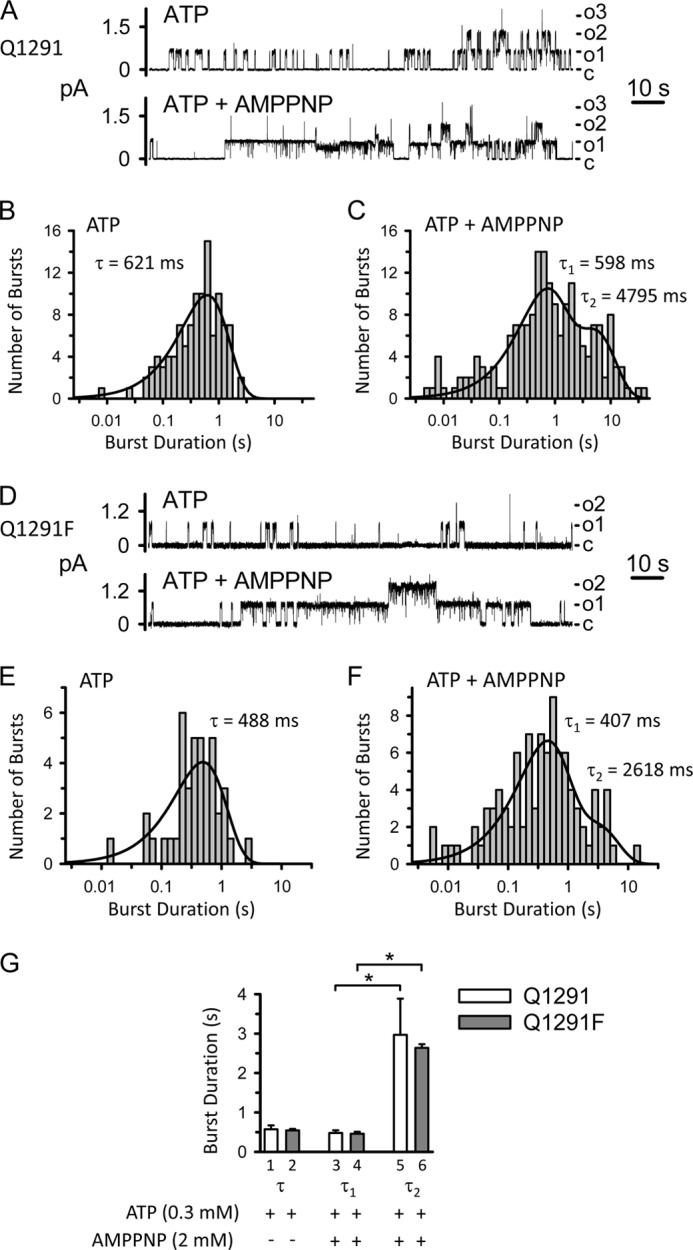
**Effect of AMPPNP on wild-type (*Q1291*; *A–C*) and Q1291F (*D–F*) CFTR burst duration.** *A* and *D*, examples of two 140-s current traces from the same excised membrane patch containing at least three wild-type (*A*) and two Q1291F (*D*) CFTR channels before and after adding AMPPNP (2 mm) to the cytosolic surface. ATP (0.3 mm) and PKA catalytic subunit were present throughout the experiments. *c*, channel closed state; *o1*, *o2*, and *o3*, open states. Holding voltage was −80 mV. For illustration purposes, traces were digitally low pass-filtered at 50 Hz. *B*, *C*, *E*, and *F*, burst duration histograms of wild-type (*B* and *C*) and Q1291F (*E* and *F*) CFTR channel activity before (*B* and *E*) and after (*C* and *F*) adding AMPPNP derived from the experiments shown in *A* (*histograms B* and *C*) and *D* (*histograms E* and *F*). Data were plotted and fit as described under “Experimental Procedures.” *Solid lines*, superimposed fits of sums of exponential probability functions. In *B* and *E*, data were fit to single exponential functions with time constant τ. Fits with two exponential components were not statistically better. In *C* and *F*, data were fit to two-component exponential functions (with time constants τ_1_ and τ_2_), which fit the data statistically better than single exponential functions. Three exponential components did not fit the data statistically better than two components. Thus, the burst duration distributions indicated two distinct populations of bursts. *G*, summary data. Each *column* is the mean ± S.E. (*error bars*) of 3–4 experiments performed as illustrated in *A–F* with different membrane patches. Burst duration histograms in the presence of ATP but in the absence of AMPPNP were fit to single exponential functions with time constant τ as illustrated in *B* and *E*. When AMPPNP was also present, data were fit to two-component exponential functions with time constants τ_1_ and τ_2_ as illustrated in *C* and *F*. *, *p* < 0.05 compared with the results for τ_1_ (Kruskal-Wallis one-way ANOVA on ranks followed by Dunn's method of multiple comparisons *versus* control group). The differences between *columns 1–4* (one-way ANOVA) and between *columns 5* and *6* (Student's *t* test) are not significant.

To further probe ATPase-dependent gating of Q1291F CFTR, we studied the effect of adenosine 5′-(β,γ-imido)triphosphate (AMPPNP). AMPPNP is a non-hydrolyzable ATP analogue that increases wild-type CFTR current by delaying channel closing and inducing prolonged open bursts (40, 49–51). Previous work comparing burst duration histograms of wild-type CFTR in the presence of ATP alone *versus* ATP plus AMPPNP showed that in the absence of AMPPNP, there was only one population of bursts. However, after AMPPNP was added, a second population of very long bursts appeared, consistent with AMPPNP competing with ATP and preventing channel closure (40). Therefore, we predicted that AMPPNP would induce prolonged open bursts of Q1291F CFTR if channel closing was coupled to ATP hydrolysis. Experimental testing showed that we indeed detected a second population of very long Q1291F CFTR bursts after adding AMPPNP. The mean durations of the different populations of bursts were similar to those of wild-type CFTR (Fig. 6).

Consistent with our findings, a previous study showed that the Q1291A mutation did not change burst duration, interburst interval, or the ATP dose-response curve (52). Together with those results, our data suggest that Gln-1291 mutations do not substantially alter the interaction of CFTR with ATP and ATPase-dependent gating.

##### Gln-1291 Mutations Disrupt the Effects of AMP and AMP-NH_2_ on CFTR Current

**FIGURE 7.**
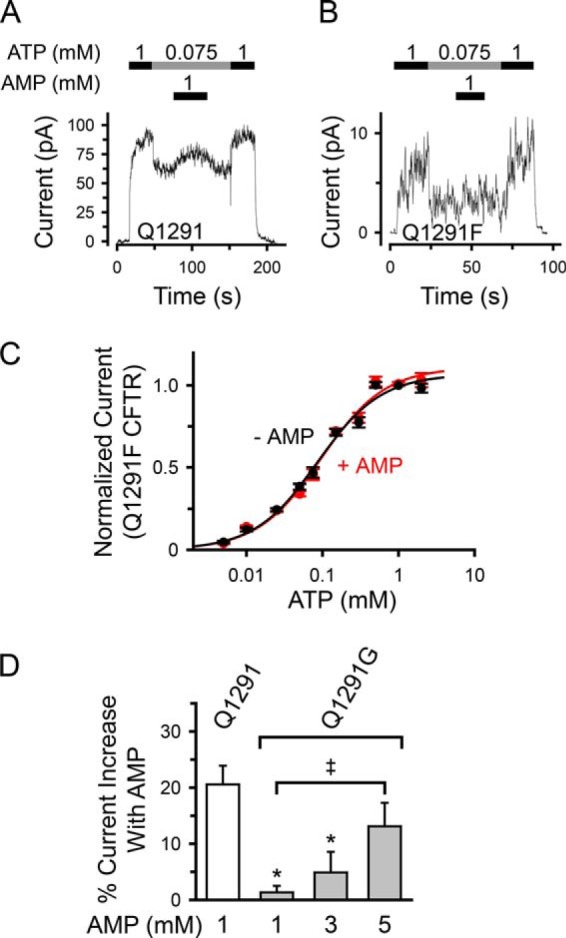
**Influence of Gln-1291 mutations on the effect of AMP on CFTR Cl^−^ current.** *A* and *B*, time courses showing the effect of AMP added at 75 μm ATP on wild-type (*Q1291*) (*A*) *versus* Q1291F (*B*) CFTR Cl^−^ current. Recordings (100 ms averages) are from excised inside-out membrane patches containing multiple wild-type (*A*) or Q1291F (*B*) CFTR channels. ATP and AMP were present during times and at concentrations indicated by *bars*. ATP was added together with PKA catalytic subunit as described under “Experimental Procedures.” Holding voltage was −40 mV. *C*, quantitative data showing lack of effect of AMP (1 mm) on ATP-dependent Q1291F CFTR current. Experiments were performed as shown in *B*. Data are from 15 patches with *n* ≥ 8 for each ATP concentration. Current measurements in the absence of AMP are the same as shown in Fig. 4 for Q1291F CFTR. All current recordings were normalized to the current obtained with 1 mm ATP in the absence of AMP. *Lines* are fit to the Hill equation. Data in the absence of AMP were fit using an apparent *K_m_* of 87 ± 7 μm, maximum normalized current at high ATP concentrations of 1.07 ± 0.03, and a Hill coefficient of 1.04 ± 0.07. Data in the presence of AMP were fit using an apparent *K_m_* of 96 ± 9 μm, maximum normalized current at high ATP concentrations of 1.11 ± 0.03, and a Hill coefficient of 1.03 ± 0.07. *D*, removal of the Gln-1291 side chain (Q1291G mutation) reduces the potency of AMP to increase CFTR Cl^−^ current. The percentage of current increase with AMP was calculated as the difference between the current at 75 μm ATP before and after adding AMP, divided by the current in the absence of AMP (at 75 μm ATP) and multiplied by 100. Experiments were performed as shown in *A*. Each *column* shows the mean ± S.E. (*error bars*) of 7–13 individual experiments obtained from at least three membrane patches. *, *p* < 0.05 compared with wild type (Kruskal-Wallis one-way ANOVA on ranks followed by Dunn's method of multiple comparisons *versus* control group). *Double dagger*, *p* < 0.05 (Mann-Whitney rank sum test).

**FIGURE 8.**
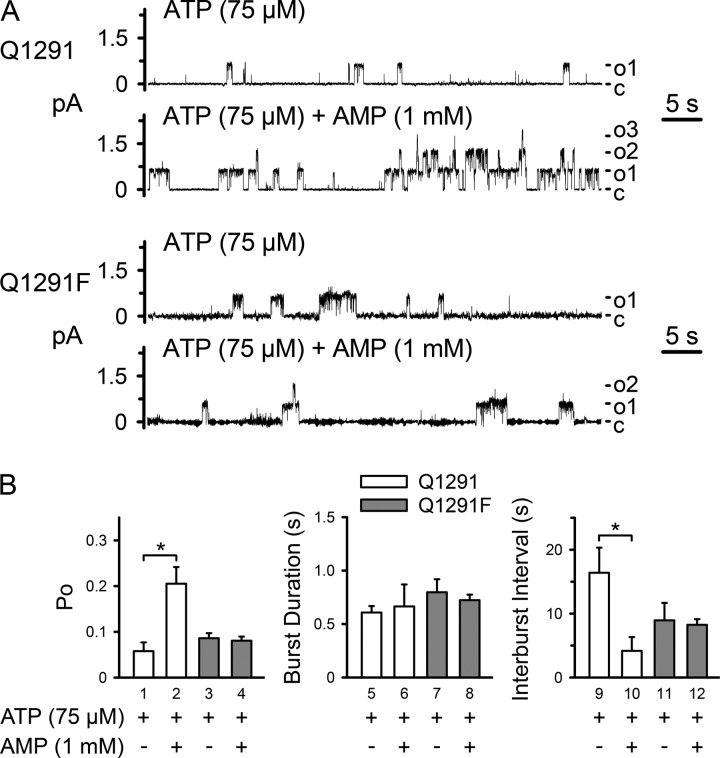
**Effect of AMP on gating of wild-type (*Q1291*) and Q1291F CFTR.** *A*, examples of two 1-min current recordings from the same excised membrane patch, each containing at least three wild-type or Q1291F CFTR channels, before and after adding AMP (1 mm) to the cytosolic surface. ATP (75 μm) and PKA catalytic subunit were present throughout the experiments. Holding voltage was −80 mV. *c*, channel closed state; *o1*, *o2*, and *o3*, open states. For illustration purposes, traces were digitally low pass-filtered at 50 Hz. *B*, average single channel properties. Data are means ± S.E. (*error bars*) of six wild-type and three Q1291F CFTR patches. *, *p* < 0.05 (Wilcoxon signed rank test). No significant differences were detected between *bars 1*, *3*, and *4*, between *bars 5–8*, and between *bars 9*, *11*, and *12* (Kruskal-Wallis one-way ANOVA on ranks).

**FIGURE 9.**
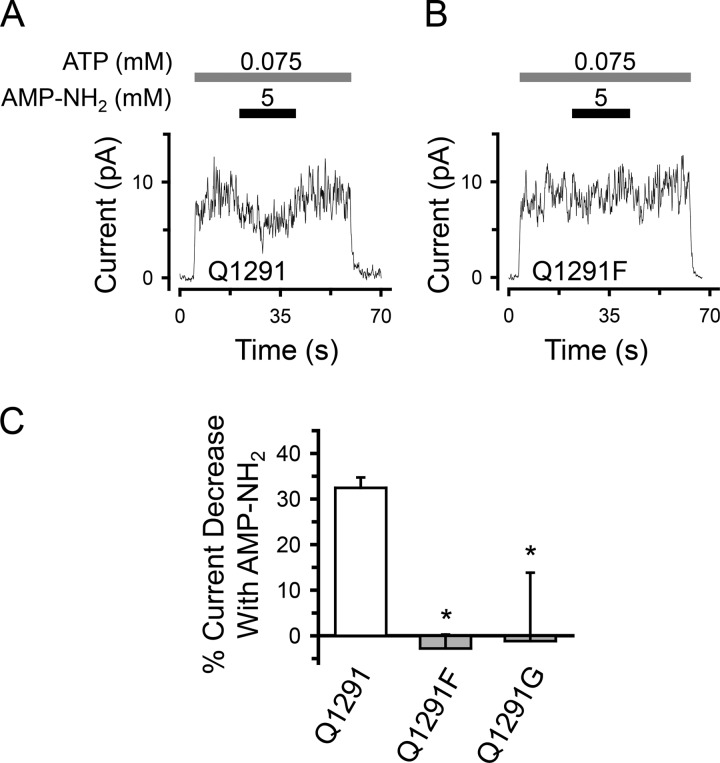
**Effect of Gln-1291 mutations on AMP-NH_2_ inhibition of CFTR Cl^−^ current.** *A* and *B*, time courses showing the effect of AMP-NH_2_ added at 75 μm ATP on wild-type (*Q1291*) (*A*) *versus* Q1291F (*B*) CFTR Cl^−^ current. Recordings (100 ms averages) are from excised inside-out membrane patches containing multiple wild-type (*A*) or Q1291F (*B*) CFTR channels. ATP and AMP-NH_2_ were present during the times and at the concentrations indicated by *bars*. ATP was added together with PKA catalytic subunit as described under “Experimental Procedures.” Holding voltage was −50 mV. *C*, quantitative data. Experiments were performed as shown in *A* and *B* with [ATP] = 75 μm. The percentage of current decrease with 5 mm AMP-NH_2_ was calculated as described in the legend to Fig. 3*B* for Ap_5_A. *Columns* show the means ± S.E. (*error bars*) of 15 (wild type), 11 (Q1291F mutant), and 5 (Q1291G mutant) individual experiments obtained from two (wild type) and three (Q1291F and Q1291G mutants) membrane patches. *, *p* < 0.001 compared with wild-type (Kruskal-Wallis one-way ANOVA on ranks followed by Dunn's method of all pairwise multiple comparisons).

We predicted that if Gln-1291 mutations interfered with the interaction of Ap_5_A at the AMP-binding site, these mutations would alter the effects of AMP and AMP-NH_2_ on CFTR current. Previous studies demonstrated that both nucleotides bound the AMP-binding site of CFTR, but they altered CFTR current differently. AMP changed the shape of the ATP dose-response curve of CFTR by inducing a pattern of positive cooperativity for ATP. As a result, it induced a current increase at lower ATP concentrations, whereas it did not change maximal current at high ATP concentrations (15). AMP-NH_2_ is an AMP analogue that cannot act as a phosphoryl group acceptor. AMP-NH_2_ partially inhibited CFTR current non-competitively with ATP (15, 53). Consistent with this earlier work, we found that AMP increased (Figs. 7 (*A* and *white bar* in *D*) and 8) and AMP-NH_2_ decreased (Fig. 9, *A* and *white bar* in *C*) wild-type CFTR current in the presence of 75 μm ATP. In contrast, no change in current was observed when Gln-1291 was replaced by phenylalanine or glycine (Figs. 7 and 9). Because the effect of AMP on wild-type CFTR current depended on the ATP concentration (15), we tested its effect on Q1291F CFTR current at a range of different ATP concentrations. AMP did not affect Q1291F CFTR current at any ATP concentration and thus did not alter its ATP dose-response curve (Fig. 7*C*). We also examined the effects of AMP on channel gating at 75 μm ATP (Fig. 8). Consistent with their ATP dose-response curves (Fig. 4), wild-type and Q1291F CFTR exhibited similar open state probabilities (*P_o_*), which were lower than those obtained at 1 mm ATP (compare *P_o_* values in Figs. 5*B* and 8*B*). As reported before (15), AMP increased *P_o_* of wild-type CFTR by decreasing the interburst interval (*i.e.* by increasing the rate of opening into a burst). In contrast, AMP had no effect on Q1291F CFTR gating. These results are consistent with the disruption of nucleotide interactions at the AMP-binding site in Q1291F CFTR. If the Gln-1291 side chain interacted with AMP as predicted based on the SMC-NBD structure (Fig. 1) (19), then removal of this side chain (Q1291G mutation) should affect the interaction of AMP with CFTR. We found that AMP induced some current increase but only at high AMP concentrations (Fig. 7*D*), consistent with reduced affinity.

##### The Q1291F Mutation Disrupts Photolabeling of the AMP-binding Site with 8-N_3_-AMP

**FIGURE 10.**
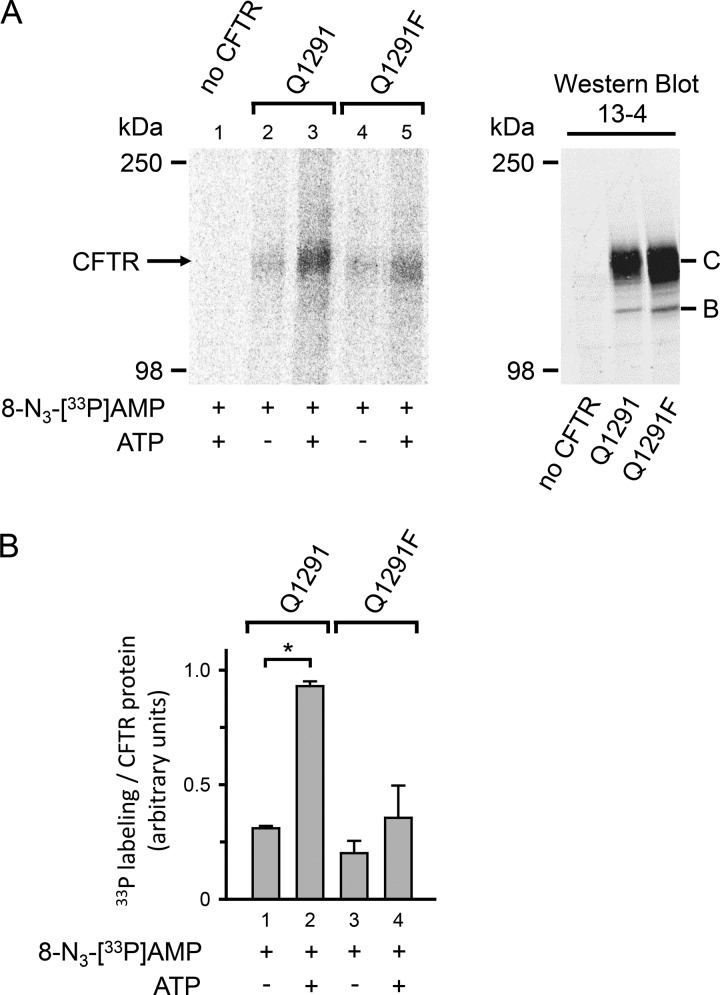
**Photolabeling of wild-type (*Q1291*) and Q1291F CFTR with 8-N_3_-[^33^P]AMP.** *A*, *left*, autoradiograph. Membranes containing 50 μg of protein from either wild-type or Q1291F CFTR-expressing HeLa cells or from HeLa cells not infected with the recombinant vaccinia virus encoding CFTR (*no CFTR*) were mixed on ice with 94 μCi of 8-N_3_-[^33^P]AMP (>3000 Ci/mmol) in the absence or presence of 8.3 mm non-radioactive ATP as indicated *below* the *lanes* of the autoradiograph. All samples were immediately irradiated with UV light for 30 s. *Right*, Western blot probed with CFTR antibody 13-4 of 50 μg of the membrane protein preparations used in the *left panel. Letters* label highly glycosylated (*C*) and core-glycosylated (*B*) CFTR. The majority of wild-type and Q1291F CFTR migrated as *band C. B*, quantitative data. Experiments were performed as illustrated in *A*. Radioactivity incorporated into the CFTR band was quantified by digital autoradiography and normalized for the amount of CFTR protein present in 50 μg of membrane protein quantified by Western blotting as described under “Experimental Procedures.” *, *p* < 0.01 (Student's paired *t* test, *n* = 3). No significant differences were detected between *bars 1*, *3*, and *4* (one-way ANOVA, *n* = 3). *Error bars*, S.E.

We hypothesized that if the Q1291F mutation abolished nucleotide interactions at the AMP-binding site, photolabeling of this site with 8-N_3_-AMP should be disrupted. In a previous study (17), we used photolabeling with 8-N_3_-[^32^P]AMP to show that membrane-embedded CFTR bound AMP at a site distinct from the ATP-binding sites. We found that 8-N_3_-[^32^P]AMP photolabeled CFTR and that labeling significantly increased in the presence of ATP. Non-radioactive AMP or Ap_5_A reduced 8-N_3_-[^32^P]AMP photolabeling in the presence of ATP but not in its absence. These results showed that only the increase in labeling signal caused by the presence of ATP represented specific labeling of the AMP-binding site (17). We expressed both wild-type and Q1291F CFTR in HeLa cells and collected cell membranes. Western blots demonstrated the presence of CFTR (Fig. 10*A*, *right*). In both cases, the majority of the CFTR protein migrated as the highly glycosylated band C (31, 35). Consistent with our earlier work, we found labeling of wild-type CFTR with 8-N_3_-[^33^P]AMP that significantly increased in the presence of ATP (compare *lanes 2* and *3*, Fig. 10*A*, *left*). We also detected a labeling signal with Q1291F CFTR. However, the ATP-dependent increase in 8-N_3_-[^33^P]AMP photolabeling was disrupted by the mutation (compare *lanes 2* and *3* with *lanes 4* and *5*, Fig. 10*A*, *left panel*, and quantitative data in Fig. 10*B*).

##### The Q1291F Mutation Disrupts CFTR Adenylate Kinase Activity

**FIGURE 11.**
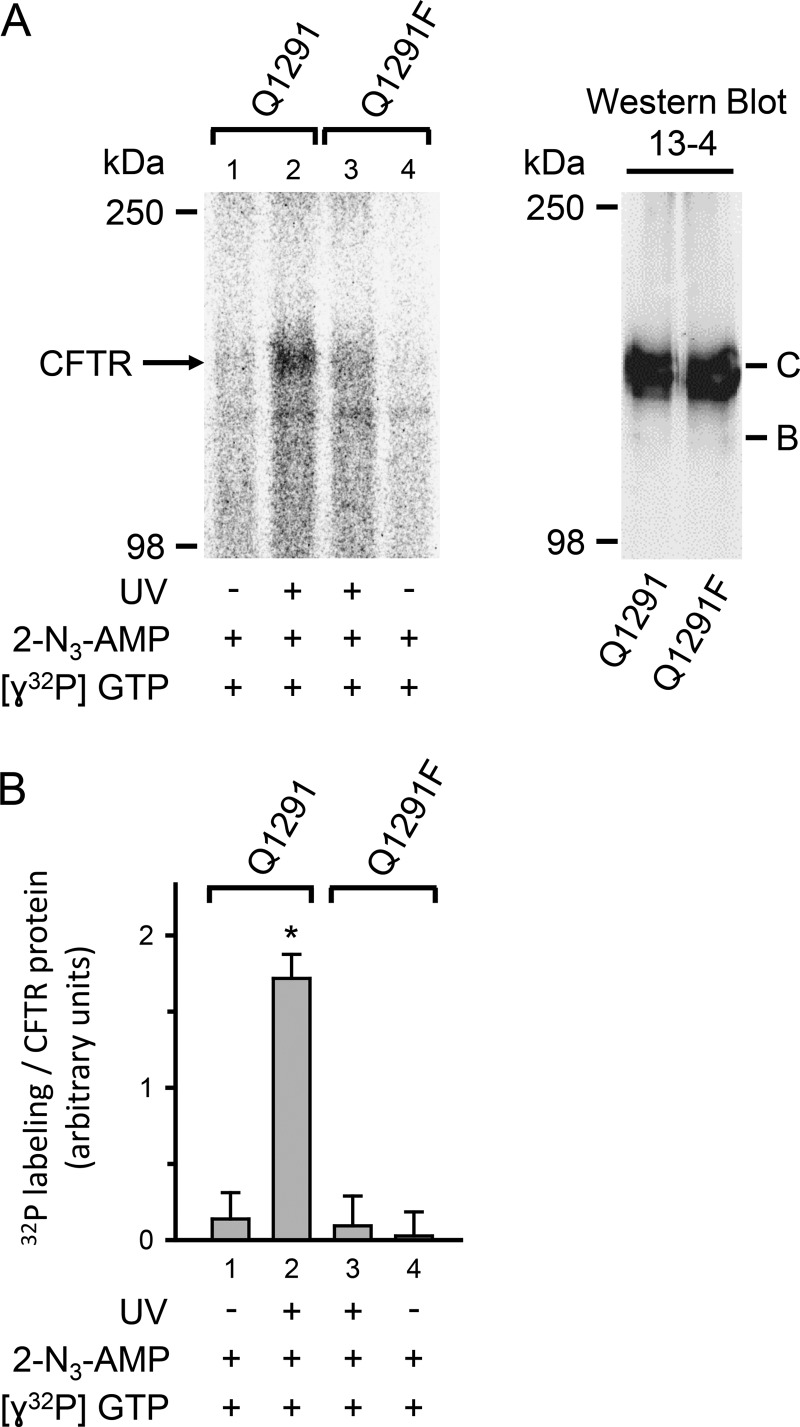
**Adenylate kinase activity of wild-type (*Q1291*) and Q1291F CFTR.** *A*, *left*, autoradiograph of immunoprecipitated wild-type and Q1291F CFTR fractionated on a 6% SDS-polyacrylamide gel. Membranes containing 50 μg of protein from either wild-type or Q1291F CFTR-expressing HeLa cells were used in each reaction. Membranes were incubated together with 50 μm non-radioactive 2-N_3_-AMP and 30 μCi of [γ-^32^P]GTP (6000 Ci/mmol) for 5 min at 37 °C followed by UV irradiation for 30 s, as indicated *below* each *lane. Right*, Western blot probed with CFTR antibody 13-4 of 50 μg of the membrane protein preparations used in the *left panel. Letters* label highly glycosylated (*C*) and core-glycosylated (*B*) CFTR. *B*, quantitative data. Radioactivity incorporated into CFTR band was quantified by digital autoradiography and normalized to the amount of CFTR protein measured by Western blot as described in the legend to Fig. 10*B*. *, *p* < 0.001 when compared with *bar 1*, *3*, or *4*. No significant differences were detected between *bars 1*, *3*, and *4* (one-way ANOVA followed by Holm-Sidak's method of all pairwise multiple comparisons, *n* = 3). *Error bars*, S.E.

The data suggest that the Q1291F mutation would interfere with adenylate kinase activity. We recently developed a biochemical assay of CFTR adenylate kinase activity and found that membrane-bound CFTR catalyzed transfer of the radioactive γ-phosphate of [γ-^32^P]GTP onto 2-N_3_-AMP, forming radioactive 2-N_3_-[β-^32^P]ADP (16). UV light then trapped the reaction product, 2-N_3_-[β-^32^P]ADP, on CFTR, because it induced photolysis of the N_3_ group and formation of a reactive intermediate that covalently attached to nearby amino acid residues (54, 55). Thus, CFTR became radioactively labeled (16). We chose to use GTP rather than ATP because it is not a substrate of protein kinases present in the membrane preparation, and thus there was decreased “nonspecific” radioactive labeling of CFTR in the absence of UV light. Work by Anderson *et al.* (56) showed that CFTR accepts GTP as well as ATP. Incubating membranes containing wild-type CFTR with [γ-^32^P]GTP and non-radioactive 2-N_3_-AMP, followed by UV irradiation, labeled CFTR (Fig. 11). In contrast, Q1291F CFTR showed very little labeling. These results indicate defective adenylate kinase activity of the mutant.

##### The Q1291F Mutation Interferes with Channel Opening in the Presence of Physiologic Concentrations of ATP, ADP, and AMP

**FIGURE 12.**
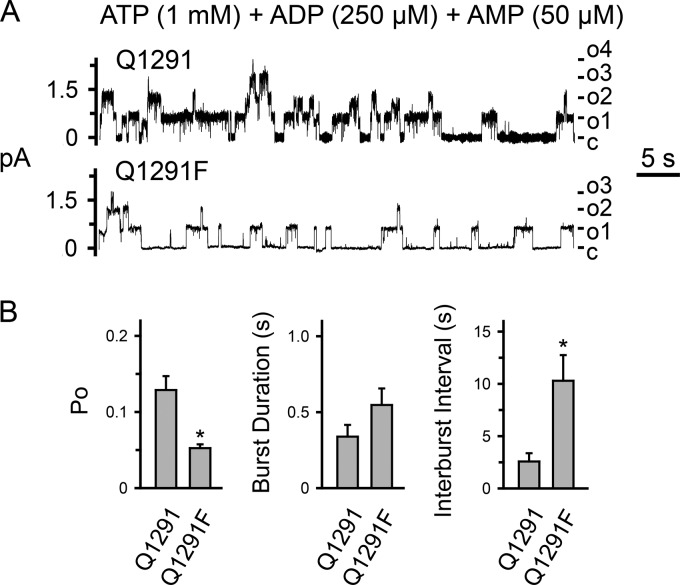
**Effect of physiologic concentrations of ATP, ADP, and AMP on gating of wild-type (*Q1291*) and Q1291F CFTR.** *A*, examples of 1-min current recordings from excised membrane patches, each containing at least four wild-type or Q1291F CFTR channels in the presence of 1 mm ATP, 250 μm ADP, and 50 μm AMP at the cytosolic surface. The PKA catalytic subunit was present throughout the experiments. Holding voltage was −80 mV. *c*, channel closed state; *o1*, *o2*, *o3*, and *o4*, open states. For illustration purposes, traces were digitally low pass-filtered at 50 Hz. *B*, average single channel properties. Data are means ± S.E. (*error bars*) of four wild-type and five Q1291F CFTR patches. *, *p* < 0.05 (Mann-Whitney rank sum test).

We examined the functional consequences of the Q1291F mutation on channel gating under physiologic conditions (*i.e.* in the presence of ATP, ADP, and AMP). ADP inhibits CFTR channel function at least in part by competing with ATP (15, 41, 57–62). CFTR adenylate kinase activity converts ADP to ATP at one of the two ATP-binding sites (15). Therefore, we hypothesized that when all three nucleotides are present, the adenylate kinase-deficient Q1291F mutant displays a lower open state probability than wild-type CFTR. We tested this hypothesis using the patch clamp technique with excised membrane patches. In the presence of 1 mm ATP, 250 μm ADP, and 50 μm AMP, Q1291F CFTR indeed displayed a significantly lower open state probability (*P_o_*) and longer closed interburst intervals (*i.e.* a reduced rate of opening into a burst) than wild-type CFTR (Fig. 12). We chose these concentrations of ATP, ADP, and AMP because they are within reported physiologic ranges (63–69).

##### The Q1291F Mutation Causes Defective CFTR Cl^−^ Channel Function in Primary Human Airway Epithelia

**FIGURE 13.**
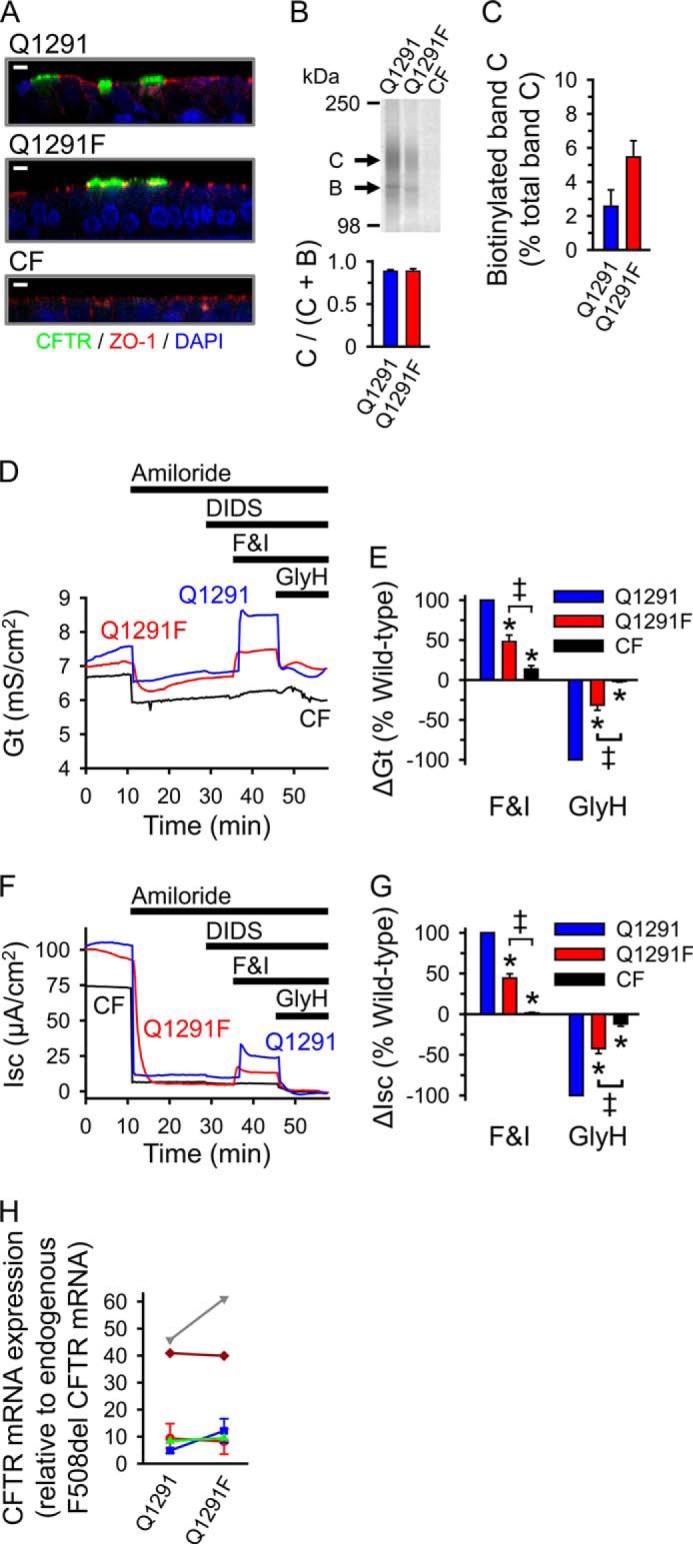
**Expression and function of wild-type and Q1291F CFTR in well differentiated primary human airway epithelia.** *A*, immunostaining of differentiated CF human airway epithelia from the same donor infected with recombinant adenovirus expressing wild-type (*Q1291*) or Q1291F CFTR and of uninfected control. CFTR appears *green*, and the tight junction protein ZO-1 appears *red*. Nuclei (DAPI-stained) are *blue. White bars*, 5 μm. *B*, *top*, Western blot probed with CFTR antibody 769 of cell lysates of differentiated CF human airway epithelia from the same donor fractionated on a 6% SDS-polyacrylamide gel. Two of these epithelia were infected with recombinant adenovirus to express wild-type or Q1291F CFTR 4 days prior to the experiment as described under “Experimental Procedures.” *Letters* label highly glycosylated (*C*) and core-glycosylated (*B*) CFTR (see the legend to Fig. 2). *Bottom*, quantitative data (means ± S.E. (*error bars*)) for the fraction of CFTR migrating as band C. CFTR protein migrating as bands B and C was quantified by Western blotting as described under “Experimental Procedures.” Depicted is the ratio of CFTR protein in band C *versus* the total CFTR protein in bands B and C. Data are from three independent experiments using airway epithelia from two different CF donors. No significant differences were detected (Student's *t* test). *C*, quantitative data (means ± S.E.) for the fraction of band C-CFTR (in percent) that bound to NeutrAvidin after exposing intact primary CF human airway epithelia cultured at the air-liquid interface (42) to apical surface biotinylation. Experiments were performed 4 days after infection with recombinant adenovirus to express either wild-type or Q1291F CFTR as described under “Experimental Procedures.” Only the apical surface was exposed to Sulfo-NHS-SS-Biotin. Data are from three independent experiments using airway epithelia from three different CF donors. No significant differences were detected (Student's *t* test). *D* and *F*, representative recordings of transepithelial conductance (*Gt*; *D*) and short circuit current (*Isc*; *F*) during an Ussing chamber study with three airway epithelia from the same CF donor. Two of these epithelia were infected with recombinant adenovirus to express wild-type or Q1291F CFTR. Sequential additions into the apical chamber were 100 μm amiloride, 100 μm DIDS, 10 μm forskolin plus 100 μm IBMX (*F&I*), and 100 μm GlyH-101 (*GlyH*), as indicated by the *bars. E* and *G*, changes in *Gt* (Δ*Gt*; *E*) and Isc (Δ*Isc*; *G*) after sequentially adding forskolin and IBMX to activate and GlyH-101 to inhibit CFTR are shown. Data were normalized to the changes in *Gt* (*E*) and *Isc* (*G*) observed in epithelia expressing wild-type CFTR during the same experiment. Values are means ± S.E. of 10 experiments performed as shown in *D* and *F* using epithelia from five different donors. *, *p* < 0.001 compared with wild type (*blue bars*); *double daggers*, *p* < 0.001 (one-way repeated measures ANOVA followed by Holm-Sidak's method of all pairwise multiple comparisons). *H*, relative expression levels of wild-type and Q1291F CFTR in primary CF human airway epithelia used in the Ussing chamber studies (*D–G*) determined by quantitative PCR as described under “Experimental Procedures.” Values for wild-type and Q1291F CFTR from epithelia of the same donor are connected by *lines* and are depicted in the *same color*.

The data suggest that the Q1291F mutation might cause defective Cl^−^ channel function in living cells. To test this hypothesis, we expressed either wild-type CFTR or the adenylate kinase-deficient Q1291F mutant in well differentiated primary human airway epithelia from cystic fibrosis patients (that lack endogenous CFTR activity) because they are closest to an *in vivo* airway epithelium. The Q1291F mutation did not reduce processing of CFTR to the apical membrane (Fig. 13, *A–C*). We studied transepithelial conductance (*Gt*) and short circuit current (*Isc*) of these epithelia in Ussing chambers. After baseline *Gt* and *Isc* had stabilized, we sequentially added amiloride to inhibit epithelial sodium channels and hyperpolarize the apical membrane, thereby generating a driving force for Cl^−^ secretion; DIDS to inhibit DIDS-sensitive non-CFTR Cl^−^ channels; forskolin and IBMX to increase cellular levels of cAMP, leading to phosphorylation and activation of CFTR; and GlyH-101 (70) to inhibit CFTR. Fig. 13, *D* and *F*, shows recordings from one experiment with epithelia from the same donor. We found that the forskolin/IBMX-induced and the GlyH-101-sensitive changes in transepithelial conductance (Fig. 13, *D* and *E*), which are directly related to CFTR channel activity, as well as the corresponding changes in short circuit current (Fig. 13, *F* and *G*) were significantly smaller in epithelia expressing Q1291F CFTR than in epithelia expressing wild-type CFTR. Although there was variability in expression levels between epithelia from different donors, expression of the mutant was not lower than wild-type CFTR expression in cells from the same donor (Fig. 13*H*). These results indicate defective channel function of Q1291F CFTR.

## Discussion

Although adenylate kinase activity has been demonstrated in CFTR both electrophysiologically (15, 62) and biochemically (16), the residues involved in the interaction of AMP and in this enzymatic activity have remained elusive. We have identified a CFTR amino acid that plays an important role in adenylate kinase-dependent gating. Notably, this residue, the Q-loop glutamine in NBD2, is highly conserved among ABC transporters.

Our results with CFTR are consistent with the crystal structure of the NBD of the non-membrane-bound ABC protein SMC in complex with Ap_5_A (19). In that structure, one adenosine, the adjacent α-, β-, and γ-phosphates of Ap_5_A, and the Mg^2+^ ion are bound in the same way as ATP in the structure of the SMC-NBD in complex with Mg-ATP (71). The second adenosine forms a stacking interaction with the carboxamide side-chain group of the conserved Q-loop glutamine. A stacking interaction of an adenine with a glutamine has also been observed in the crystal structure of eosinophil-derived neurotoxin, a member of the pancreatic RNase A family, in complex with Ap_5_A (72); Ap_5_A is a potent inhibitor of its ribonucleolytic activity (73). The studies with SMC-NBD also revealed Ap_5_A-induced conformational changes. Although ATP did not change the SMC-NBD structure, binding of Ap_5_A rotated the two lobes of the NBD by ∼15° (19). These results suggest that AMP binding to the Q-loop glutamine at the interface of the two NBD lobes and adenylate kinase activity induce distinct conformational changes in SMC. Correspondingly, previous patch clamp studies with CFTR showed that the relationship between ATP concentration and Cl^−^ current fit the Michaelis-Menten equation in the absence of other nucleotides. However, AMP induced a pattern of positive cooperativity for ATP, whereas Ap_5_A induced a pattern of negative cooperativity, consistent with conformational changes (15). The Q-loop forms extensive contacts with the transmembrane domains in ABC transporters (11, 74). Thus, it is in a position where it could be part of the coupling mechanism between the ATP catalytic cycle of the transporter and its transmembrane domains (75). With the conserved Q-loop glutamine participating in adenylate kinase-dependent gating, such an arrangement could couple adenylate kinase activity to conformational changes in the transmembrane domains. In CFTR, it could link this enzymatic activity with channel gating.

Previous structural data suggested that the Q-loop glutamine contributes its side-chain oxygen to the coordination sphere of the bound Mg^2+^ (10, 11, 13, 19, 71). Our results, however, imply that Gln-1291 does not play an essential role in the interaction of ATP with CFTR. Consistent with this conjecture, Berger *et al.* (52) had found that substituting Gln-1291 of CFTR with alanine changed neither the ATP dose-response curve nor ATP-dependent CFTR gating. Results with murine P-glycoprotein indicated that the Q-loop glutamines are not required for ATP hydrolysis. Mutating these residues to alanine or glutamate reduced but did not abolish drug-induced ATPase activity; the *K_m_* for Mg-ATP and the Mg^2+^ dependence of the ATPase activity were not altered. Furthermore, the Mg^2+^ concentration at which half-maximal ATPase activity occurred did not differ between wild type and mutants. Vanadate trapping of Mg-8-N_3_-ADP was observed in both P-glycoprotein wild-type and Q mutants, indicating that the conserved Q-loop glutamine is not an essential ligand to the Mg^2+^ in the catalytic transition state (76). In addition, a study with human MRP1 (multidrug resistance protein 1), which belongs to the same subfamily of ABC transporters as CFTR, concluded that the Q-loop glutamines do not play a significant role in ATP hydrolysis (77). Structural and functional data with CFTR and other ABC transporters rather showed that a conserved glutamate at the end of the Walker B motif is essential for ATP hydrolysis (11, 78–81). That residue hydrogen-bonds via its carboxyl group to the water molecule attacking the ATP γ-phosphate and polarizes it (11).

The interaction of the Q-loop glutamine side-chain oxygen with Mg^2+^ may stabilize the spatial orientation of the residue. Such an effect might explain, at least in part, how Mg-ATP facilitates the interaction of AMP with CFTR (17).

A loop structure being involved in the interaction of the AMP adenosine is reminiscent of the putative AMP-binding site of the human coilin-interacting nuclear ATPase protein (hCINAP). This protein was first identified as AK6 (adenylate kinase isoform 6). It shares less than 18% sequence identity with other adenylate kinase isoforms (82). Like CFTR, SMC, and Rad50, hCINAP is both an adenylate kinase and an ATPase. Its nucleotide monophosphate-binding domain contains a loop that is not observed in other adenylate kinases. Although hCINAP has not yet been crystallized in complex with AMP, induced fit docking calculations predict that the loop might contribute to binding the AMP adenosine (83).

The Q-loop glutamine is a highly conserved residue within the core structure of ABC-NBDs. Therefore, we speculate that adenylate kinase activity may be a more widespread feature of ABC proteins.

We showed that mutating the Q-loop glutamine 1291 in CFTR to phenylalanine selectively abolished adenylate kinase-dependent gating. Although this mutation did not notably affect channel function in the presence of ATP alone (*i.e.* under experimental conditions testing ATPase-dependent gating), Q1291F CFTR displayed markedly reduced channel activity in the presence of physiologic concentrations of ATP, ADP, and AMP. Consistently, its channel function in well differentiated primary human airway epithelia was significantly reduced, suggesting an important but not exclusive role of adenylate kinase-dependent gating *in vivo*. Interestingly, a mutation at this position, Q1291R, has been described in a pancreatic insufficient CF patient carrying the F508del mutation on the other chromosome (84).

Bhaskara *et al.* (18) identified a yeast Rad50 mutation causing loss of adenylate kinase activity without reducing ATPase activity. A yeast strain expressing this mutant had defects in meiosis and telomere length maintenance (18). Our results and the Rad50 studies suggest that adenylate kinase activity is important for the function of some ABC proteins.

## Footnotes

3 The abbreviations used are:

CFTR: cystic fibrosis transmembrane conductance regulator
CF: cystic fibrosis
ABC: ATP-binding cassette
AMP-NH_2_: adenosine 5′-monophosphoramidate
AMPPNP: adenosine 5′-(β,γ-imido)triphosphate
Ap_5_A: P^1^,P^5^-di(adenosine-5′) pentaphosphate
2-N_3_-[β-^32^P]ADP: 2-[β-^32^P]azidoadenosine 5′-diphosphate
8-N_3_-ADP: 8-azidoadenosine 5′-diphosphate
2-N_3_-AMP: 2-azidoadenosine 5′-monophosphate
8-N_3_-AMP: 8-azidoadenosine 5′-monophosphate
8-N_3_-[^32^P]AMP: 8-[^32^P]azidoadenosine 5′-monophosphate
8-N_3_-[^33^P]AMP: 8-[^33^P]azidoadenosine 5′-monophosphate
ANOVA: analysis of variance
DIDS: 4,4′-diisothiocyanotostilbene-2,2′-disulfonic acid
E-64: *trans*-epoxysuccinyl-L-leucylamido-(4-guanidino)butane
GlyH-101: (naphthalen-2-ylamino)-acetic acid (3,5-dibromo-2,4,-dihydroxy-benzylidene)-hydrazide
hCINAP: human coilin-interacting nuclear ATPase protein
NBD: nucleotide-binding domain
SMC: structural maintenance of chromosome
Tricine: *N*-[2-hydroxy-1,1-bis(hydroxymethyl)ethyl]glycine
IBMX: 3-isobutyl-2-methylxanthine.
